# Glucocorticoids and breast cancer risk

**DOI:** 10.1186/s12916-021-02036-y

**Published:** 2021-08-02

**Authors:** Kelly A. Hirko, A. Heather Eliassen

**Affiliations:** 1 Department of Epidemiology and Biostatistics, College of Human Medicine, Michigan State University, 909 Fee Road, East Lansing, MI 48824 USA; 2 Department of Epidemiology, Harvard T.H. Chan School of Public Health, Boston, MA USA; 3 Channing Division of Network Medicine, Department of Medicine, Brigham and Women’s Hospital and Harvard Medical School, Boston, MA USA

**Keywords:** Glucocorticoids, Breast cancer, Estrogen receptor

## Background

Glucocorticoids are essential endogenous steroid hormones secreted in response to stress, and treatment with synthetic forms is widespread for numerous inflammatory conditions including asthma, chronic obstructive pulmonary disease, inflammatory bowel disease, and arthritis [1]. Emerging evidence suggests that glucocorticoids can exert both anti- and pro-inflammatory effects, modulated through suppressing or augmenting immune response [1]. Given these divergent biologic properties, glucocorticoids could theoretically reduce breast cancer risk via anti-inflammatory effects or could increase breast cancer risk and progression by inducing insulin resistance and promoting immunosuppression [2]. Yet, despite biological plausibility, epidemiologic studies of glucocorticoid use and breast cancer are limited.

## New data

As such, the recent prospective cohort study by Cairat et al. [3] is an important contribution to the literature as an investigation into the potential role of glucocorticoids in breast cancer etiology. In this study, authors evaluated associations between glucocorticoid use and breast cancer risk among over 65,000 postmenopausal French women in the E3N cohort. Using linkage to outpatient health expenditures, authors observed that receipt of at least two reimbursements of systemic glucocorticoids was associated with a reduced risk of invasive breast cancer, which appeared to follow a dose-response trend with increasing number of reimbursements. The observed associations did not differ substantially by type of glucocorticoid, or by timing or duration of use. However, inverse associations were significant only among women older than 60 years at first use. In stratified analyses, the inverse associations were restricted to estrogen receptor (ER)-positive tumors, and stage I and II disease. Interestingly, authors also observed positive associations between glucocorticoids and risk of in situ breast cancer and stage III/IV breast cancer, although confidence intervals for these measures of association were wide given smaller numbers of cases. In sensitivity analysis, only recurrent users (not occasional users) were at higher risk of in situ or stage III or IV disease and only recurrent users were at lower risk of stage I or II breast cancer. The authors conclude that the association between systemic glucocorticoid use and breast cancer risk may differ by tumor subtypes and stage of disease.

Unlike prior studies, Cairat et al. were able assess whether the associations of glucocorticoids and breast cancer differed according to hormone receptor status, histological subtype, tumor grade, and stage at diagnosis. This is particularly important given the various pharmacological properties of glucocorticoids and evidence for cross-talk between estrogen receptors and glucocorticoid receptors in mammary epithelial cells [4], which suggests that glucocorticoids could differentially impact breast cancer subtypes. An additional strength of this study was the inclusion of comprehensive information on potential confounders including body mass index, alcohol consumption, family history of breast cancer, and use of nonsteroidal anti-inflammatory medication; factors which were not considered in prior population registry-based studies in Denmark [5, 6] where no associations between receipt of glucocorticoid prescription and breast cancer risk were observed.

This study is informative in teasing apart heterogeneity in glucocorticoid-breast cancer associations by hormone receptor status to isolate the potential impact of estrogen pathways. As authors of this study note, estrogen inhibition induced by glucocorticoids may explain the lower risk of ER-positive tumors observed in this study. Moreover, given that ER-positive tumors tend to be less aggressive than ER-negative tumors, the inverse associations observed for early stage disease may reflect the preponderance of ER-positive tumors in this group. Systemic glucocorticoid use also affects immune system function and insulin sensitivity, which could induce adipose aromatase activity and estrogen production and also directly stimulate breast cancer cell growth and invasion [7], thereby facilitating breast cancer progression and later stage of disease at diagnosis. However, biological plausibility for the observed positive association between glucocorticoids and in situ disease is less clear. Although a possible explanation for this finding may be increased surveillance for women prescribed glucocorticoids, the results were similar when restricted to women with a recent mammogram. Further, that the increased risk of in situ disease corresponds with findings for later, but not earlier, stage invasive disease, is not likely to be explained by surveillance bias, but also complicates the biological plausibility of these findings. The conflicting findings in this study underscore the need for continued research to elucidate the complex interactions between chronic inflammation, obesity, and insulin resistance in breast cancer.

## Impact and implications

The potential impact of these findings are substantial, given the widespread and increasing use of glucocorticoids over the past several decades [8, 9], and the extensive global breast cancer burden with an estimated 2.3 million new cases diagnosed in 2020 [10]. However, it should be emphasized that research on the impact of glucocorticoids and breast cancer risk is still in a nascent stage. Indeed, this is the first study to evaluate whether the relationship between glucocorticoids and breast cancer differs according to hormone receptor status, histology, and tumor stage. Thus, results need to be confirmed in large epidemiologic studies with detailed information on tumor characteristics to address the small number of in situ, ER-negative, and late stage breast cancers included in the current study. If findings are confirmed in future studies, clinical implications will also need to consider both the risks and benefits of glucocorticoid use in clinical care.

## Conclusions

In conclusion, this study’s findings are intriguing and merit follow-up to further characterize the potential role of glucocorticoids and other anti-inflammatory therapies in breast cancer etiology. Specifically, future studies recognizing the complex interplay between inflammation and adaptive immune response in breast cancer are needed to inform our understanding of etiology and the development of personalized medicine approaches in breast cancer.

## Data Availability

Not applicable
